# Oral prophylaxis for the reduction of interdental dysbiosis-associated red complex pathogens during pregnancy: a randomized clinical trial

**DOI:** 10.3389/fmed.2026.1798508

**Published:** 2026-04-23

**Authors:** Florence Carrouel, Aida Kanoute, Daouda Faye, Maryem Rhanoui, Romain Lan, Denis Bourgeois

**Affiliations:** 1 Laboratory Health Systemic Process (P2S), UR4129, University Claude Bernard Lyon 1, University of Lyon, Lyon, France; 2 Public Health Service, Department of Dentistry, Cheikh Anta Diop University, Dakar, Senegal; 3 Directorate General of Health, Ministry of Health, Dakar, Senegal; 4 Laboratory ADES, Aix Marseille University, CNRS, EFS, Marseille, France

**Keywords:** biofilm, gingival health, maternal health, microbiome, oral hygiene, periodontal pathogens, pregnancy

## Abstract

**Introduction:**

Periodontal disease during pregnancy is associated with adverse outcomes such as preterm birth, low birth weight, and preeclampsia. Socransky’s red complex pathogens including *Porphyromonas gingivalis, Tannerella forsythia,* and *Treponema denticola,* are key risk factors for these complications. This study aims to evaluate whether daily use of calibrated interdental brushes, in addition to conventional tooth brushing, reduces the bacterial load these pathogens in pregnant women.

**Methods:**

A randomized controlled trial was conducted in six obstetric clinics in Senegal between March 2022 and January 2023. One hundred pregnant women, aged 18–40 and in their third month of pregnancy, were randomized into a test group using interdental brushes plus tooth brushing and a control group using tooth brushing alone. The outcome was the change in bacterial load from the third to eighth month of pregnancy, quantified using real-time polymerase chain reaction techniques.

**Results:**

By the eighth month, the test group demonstrated a statistically significant reduction in total bacterial load compared to the control group, with a mean reduction of 36.6% (95% CI: 31.2–41.1%, *p* < 0.001). Notably, *T. denticola* load decreased by 92.1% (95% CI: 87.1–95.6%, *p* < 0.001) in the test group, while it increased in the control group. Reductions were also observed for *P. gingivalis* and *T. forsythia*, although these differences were not statistically significant (*p* = 0.061 and *p* = 0.148, respectively).

**Discussion:**

These findings suggest that adding calibrated interdental brushes to a conventional tooth brushing routine is more effective in lowering the bacterial load of harmful red complex pathogens in pregnant women.

**Conclusion:**

The results support the inclusion of interdental cleaning in prenatal oral health care guidelines as a strategy for managing periodontal risk during pregnancy.

## 1 Introduction

A balanced periodontal microbiome is essential for maintaining a healthy pregnancy (1, 2). Disruptions in the oral microbiome’s composition are associated with an increased risk of complications such as preterm birth, low birth weight, and preeclampsia. Pregnancy-related physiological changes, particularly elevated progesterone and estrogen levels, significantly impact oral health (3). These hormonal shifts increase vascular permeability, enhance blood flow to gingival tissues, and amplify inflammatory responses, making pregnant women more susceptible to periodontal inflammation (4). The heightened inflammatory response creates an environment favoring the proliferation of periodontal pathogens, especially those from Socransky’s red complex (*P. gingivalis, T forsythia,* and *T. denticola*) (5). These pathogens not only exacerbate local inflammation but also contribute to systemic inflammatory pathways, which are linked to adverse pregnancy outcomes.

The relationship between periodontal health and systemic conditions is bidirectional (6). On one hand, pregnancy-induced inflammation and hormonal changes exacerbate periodontal infections, increasing bacterial load and dysbiosis. On the other hand, periodontal pathogens, particularly those from Socransky’s red complex, can disseminate into the bloodstream, triggering systemic inflammation that may negatively impact maternal and fetal health (7). This bidirectional interaction underscores the critical importance of managing periodontal health during pregnancy to break this cycle and mitigate associated risks.

Pregnant women represent a uniquely vulnerable population due to the interplay of hormonal, physiological, and immunological changes. Elevated progesterone and estrogen levels favor microbial dysbiosis and increase gingival inflammation, while immune adjustments suppress bacterial clearance, allowing periodontal pathogens to proliferate unchecked (8). These combined factors not only exacerbate local inflammation but also increase the systemic dissemination of periodontal pathogens, triggering the release of inflammatory markers such as IL-6 and CRP.

While interdental hygiene aims to reduce dysbiosis, the three bacteria of the Socransky’s red complex should be studied in depth because of their strong association with adverse pregnancy outcomes. Previous research has demonstrated that interdental cleaning significantly reduces the bacterial load of Socransky’s red complex pathogens associated with dysbiosis, while bacteria from the green, blue, yellow, or violet complexes, which are linked to symbiosis, remain unaffected or may even increase (9). Reducing red complex pathogens is crucial for managing both periodontal and systemic risks during pregnancy, as they are resistant to standard oral hygiene practices and play a key role in severe periodontal inflammation.

Interdental spaces, as ecological niches protected by the gingival papilla, are particularly prone to biofilm accumulation and colonization by these pathogens, making them difficult to clean with conventional tooth brushing (10, 11). Studies have demonstrated a significant bacterial load in the interdental spaces of adolescents (11), young healthy adults (12), and pregnant women (13), even in the presence of an intact periodontium. For instance, a study reported an average of 10^9.11^ bacterial colonies per interdental space in women at 3 months of pregnancy. Pathogens from Socransky’s red complex were consistently found together in these biofilms (11–13), underscoring the importance of implementing effective management strategies.

Calibrated interdental brushes (IDBs) are specialized oral hygiene tools designed to efficiently clean interdental spaces (14). These brushes provide a precise cleaning solution, tailored to the unique size of each interdental space, ensuring optimal oral care. Daily use of calibrated interdental brushes, introduced early in pregnancy, significantly reduced gingival bleeding with rapid and sustained effects (15). While studies in healthy young adults have demonstrated that daily use of IDBs reduces the load of Socransky’s red complex (9), restores symbiosis, and decreases local inflammation (16), pregnant women represent a distinct population with unique hormonal, physiological, and immunological changes that significantly amplify their susceptibility to periodontal pathogens (17). These differences necessitate specific investigations to evaluate the effectiveness of IDBs in managing periodontal health and mitigating systemic risks in this vulnerable group.

Previous meta-analyses have demonstrated improvements in pregnancy outcomes following professional periodontal treatment (18–20). However, these studies primarily focus on curative interventions. Our approach is complementary, targeting interdental biofilms early in pregnancy to prevent the development of periodontitis (13). This preventive strategy is particularly relevant in regions with limited access to professional periodontal care, such as sub-Saharan Africa.

This randomized controlled trial (RCT) aims to address the gap in evidence by evaluating the efficacy of daily IDB use, in addition to conventional tooth brushing, in reducing the bacterial load of Socransky’s red complex in pregnant women between the third and eighth months of gestation. This microbiological analysis represents a secondary outcome of the OP-PE trial and should be interpreted in the context of the primary cluster randomized controlled trial (21). By improving periodontal health, this study seeks to explore whether enhanced interdental hygiene can mitigate systemic inflammation and reduce the risks of adverse pregnancy outcomes, providing an accessible and effective strategy for maternal and fetal health.

## 2 Materials and methods

### 2.1 Study design

This study was a multicenter, single-blind RCT with two parallel arms (1:1). Participants were enrolled and monitored at six Senegalese antenatal obstetric clinic: Philippe Senghor (Dakar, Senegal), Ngor (Dakar, Senegal), Nabil Choucair (Dakar, Senegal), Mame Abdou Aziz Sy (Dakar, Senegal), Gaspard Camara (Dakar, Senegal) and Mamadou Diop (Dakar, Senegal). Written informed consent was obtained from each woman before enrolment. The protocol was registered at ClinicalTrials.gov (NCT04989075) and approved by the ethical committee of Dakar (Senegal) reviewed and approved the clinical trial protocol (protocol 000086/MSAS/CNERS/SP approved on 8 June 2021). The RCT was conducted in compliance with the principles of the Declaration of Helsinki and follows the CONSORT guidelines.

### 2.2 Participants

Population was ambulatory 3 months pregnancy women who had voluntarily presented at the hospital for the first obstetrical visit between March and August 2022.

Eligibility was restricted to women from sub-Saharan Africa, aged 18–40 years, who were nulliparous at the time of the obstetrical consultation, up to 12 weeks pregnant, with no systemic or infectious diseases, no regular use of interdental cleaning tools, and no history of orthodontic or periodontal treatments.

The exclusion criteria were pregnant women with fetal distress, congenital uterine and vaginal abnormalities, infectious or systemic diseases, premature termination of pregnancy for medical reasons, periodontal lesions of stage II, III, IV (i.e., PD ≥ 4 mm, and/or CAL ≥ 4 mm), generalized (>30% of sites), history or treatment of PD, a course of dental or orthodontic treatment, absence of the 4 premolar–molar pairs, less than 20 natural teeth, excluding third molars, medication affecting the gum and/or oral mucosa, regularly using IDBs and/or dental floss and/or mouthwash, and (xi) unable to answer questions or non-cooperative.

### 2.3 Randomization and masking

Eligible participants were randomized (1:1) into either the test group (IDBs use) or the control group (usual oral hygiene practices). Randomization was stratified by site and implemented using a centralized computerized system with a random number generator to ensure allocation concealment. Investigators, laboratory personnel, and statisticians were blinded to group assignments.

Stratified randomization was employed to ensure balance in baseline characteristics between groups. Participants were stratified across the six participating antenatal clinics to account for potential site-related differences in participant demographics and clinical practices.

Randomization was conducted using a centralized computerized system, employing block randomization within each stratum to maintain equal group sizes. Allocation concealment was ensured to prevent selection bias. A total of 330 pregnant women meeting the inclusion criteria were initially enrolled in the larger study assessing preeclampsia risks. For the microbiological sub-study, a predefined sample of 100 participants was specified in the original OP-PE protocol based on an *a priori* power calculation for bacterial load outcomes. After randomization in the parent trial, participants were prospectively included using a systematic allocation procedure: within each study site and randomized arm, every third consecutively enrolled participant was selected for microbiological analysis. This approach was applied independently of clinical, periodontal, or obstetrical characteristics. Stratification by study site and treatment allocation was preserved to maintain representativeness of the parent cohort. These women were evenly distributed into the test group (*n* = 50) and the control group (*n* = 50).

Attrition rates across study periods (T1, T2, T3) were documented, with detailed reasons for loss provided, ensuring transparency in the analysis.

### 2.4 Intervention

The test group received a calibrated IDB kit (Curaprox CPS; Curaden Kriens, Switzerland) tailored to their interdental space diameters (14). Participants from the test group were instructed to perform interdental cleaning once daily (evening) with the IDBs by periodontists during the baseline visit. These participants recorded their daily use of interdental brushes in individual logbooks to monitor adherence throughout the study.

Participants in control group received no instruction. At each monthly visit, adherence in the intervention group was assessed and reinforced, whereas the control group received routine follow-up without specific interdental hygiene reinforcement. Compliance was assessed using self-reported daily logbooks reviewed at each monthly follow-up visit; no objective digital tracking devices were used.

### 2.5 Data collection and sampling

Interdental biofilm samples were collected at four standardized interdental sites (15–16, 25–26, 35–36, 45–46) at 3 months of pregnancy (baseline: T0), 4 months (T1), 6 months (T2), and 8 months of pregnancy (T3). If one of the interdental sites was missing due to the absence of a tooth, the adjacent medial site was sampled as a replacement. The appropriate IDBs (Curaden, Kriens, Switzerland) were determined during the clinical assessment of the interdental spaces (14). Selected teeth were isolated using sterile cotton rolls. Interdental biofilm was collected using a sterile, calibrated interdental brush (IDB) introduced and then removed from the interdental space (12). The 4 IDBs from one pregnant woman were pooled into 1 sterile 1.5 mL microcentrifuge tube and stored at 4 °C for further processing.

Total DNA was extracted from the IDBs using the QIAcube HT Plasticware and Cador Pathogen 96 QIAcube HT Kit (Qiagen, Hilden, Germany), following the manufacturer’s guidelines. The quantity and quality were evaluated using UV at 260 and 280 nm. The DNA concentration of each sample was measured using a NanoDrop spectrophotometer, and all samples were diluted to a final concentration of 50 ng/μL. In each reaction, 2 μL of the normalized DNA was used. Quantitative real-time PCR quantified total bacterial load and the specific pathogens *P. gingivalis, T forsythia,* and *T. denticola*. To ensure consistency, all laboratory procedures were performed by blinded technicians following a standardized protocol (12).

### 2.6 Outcomes

The outcomes presented in this article correspond to the secondary outcomes of the OP-PE trial (22), focusing on the number of pathogenic bacteria in the interdental microbiota during the pregnancy. The outcome measures were changes in the pathogenic bacterial load in interdental microbiota samples between T0 (3 months of pregnancy) and T1 (4 months of pregnancy) or T2 (6 months of pregnancy) or T3 (8 months of pregnancy).

### 2.7 Statistical analysis

Sample-size calculations were performed using the freeware STPLAN (Version 4.5, Department of Biomathematics, University of Texas M. D. Anderson Cancer Center, USA). The sample size was based on a minimal bacterial load difference of 1.30 log_10_ copies/mL between the control and IDB groups, with a common standard deviation of 2 log_10_ copies/mL, a power of 0.9, and a unilateral type I error rate of 5%. Considering an anticipated attrition rate of 10%, it was calculated that at least 50 subjects per group were required to ensure 45 participants per group complete the study.

Analyses were conducted on an intention-to-treat (ITT) basis. Missing data were assessed in both groups, and Missing Completely at Random (MCAR) test was performed to determine whether the missing data were MCAR. The results of MCAR test indicated that the missing data were MCAR (Supplementary Table S1). Multiple imputation by chained equations, based on a Monte Carlo and Markov chain algorithm under the assumption of random missing data, was applied. Imputation did not significantly alter the study outcomes. A sensitivity analysis was performed using four imputation methods: Complete Case Analysis, Mean Imputation, k-Nearest Neighbors (k = 5), and Multiple Imputation by Chained Equations (MICE). Each method was applied to assess the robustness of the results (Supplementary Tables S2–S5). MICE, based on iterative modeling, was used as the primary imputation strategy due to its ability to account for uncertainty and preserve data structure. Per-protocol analyses were also performed for participants with complete data across all time points.

Bacterial load was quantified and subsequently transformed using the log10 scale prior to statistical analysis. This transformation was performed to normalize the distribution of microbial count data and to stabilize variance, given that bacterial populations often span several orders of magnitude. The average percentage reduction in bacterial load was calculated using log₁₀-transformed values. Percentage changes were calculated from log10-transformed values and should be interpreted as relative variations on a logarithmic scale rather than direct linear percentage reductions in bacterial counts. Because bacterial loads were analyzed on a log10 scale, a reduction of 1 log10 corresponds to a tenfold decrease in bacterial count.

Normality assumptions were systematically assessed. As data distributions were not consistently normal despite log10 transformation, nonparametric tests (Mann–Whitney U and Wilcoxon signed-rank tests) were applied in addition to mixed-effects linear models to ensure robust inference.

Key statistical methods included (i) Wilcoxon signed-rank test with Bonferroni correction for changes over time (e.g., T0–T1, T1–T2), (ii) Wilcoxon signed-rank test with Bonferroni correction for changes over time (e.g., T0–T1, T1–T2) within-group comparisons; (iii) Mann–Whitney U test for differences at each time point between-group comparisons and, (iv) Mixed-effects linear models with bacterial load as the dependent variable, group (IDB/control) as the fixed effect, and participant as a random effect to account for repeated measures for longitudinal analysis. Baseline differences between groups were assessed descriptively. Although some variables showed statistical differences at baseline, these remained within clinically comparable ranges. Longitudinal mixed-effects models primarily focused on within-participant changes over time and between-group trajectory differences, thereby reducing the influence of minor baseline imbalances on the interpretation of microbiological outcomes. Given the predefined hypothesis that calibrated IDBs would lead to a reduction in bacterial load, a one-tailed test was applied to focus on detecting this expected directional effect. However, two-tailed sensitivity analyses for between-group comparisons were additionally performed.

Bonferroni correction was applied to within-group longitudinal comparisons across time points (T0–T1, T1–T2, T2–T3) to control for inflation of type I error related to repeated testing. Between-group comparisons were interpreted in the context of predefined microbiological objectives and were additionally supported by mixed-effects linear models accounting for repeated measures. No global adjustment across bacterial species was applied, as these outcomes were biologically related but distinct; therefore, secondary findings should be interpreted with appropriate caution.

Statistical significance was set at *p* < 0.05. 95% CIs were calculated from the estimates of mean bacterial load reductions and linked standard deviations. Analyses were performed using Python 3.10. Results were reported as means with 95% confidence intervals or medians with interquartile ranges, depending on data distribution.

## 3 Results

### 3.1 Characteristics of participants

**Figure 1:**
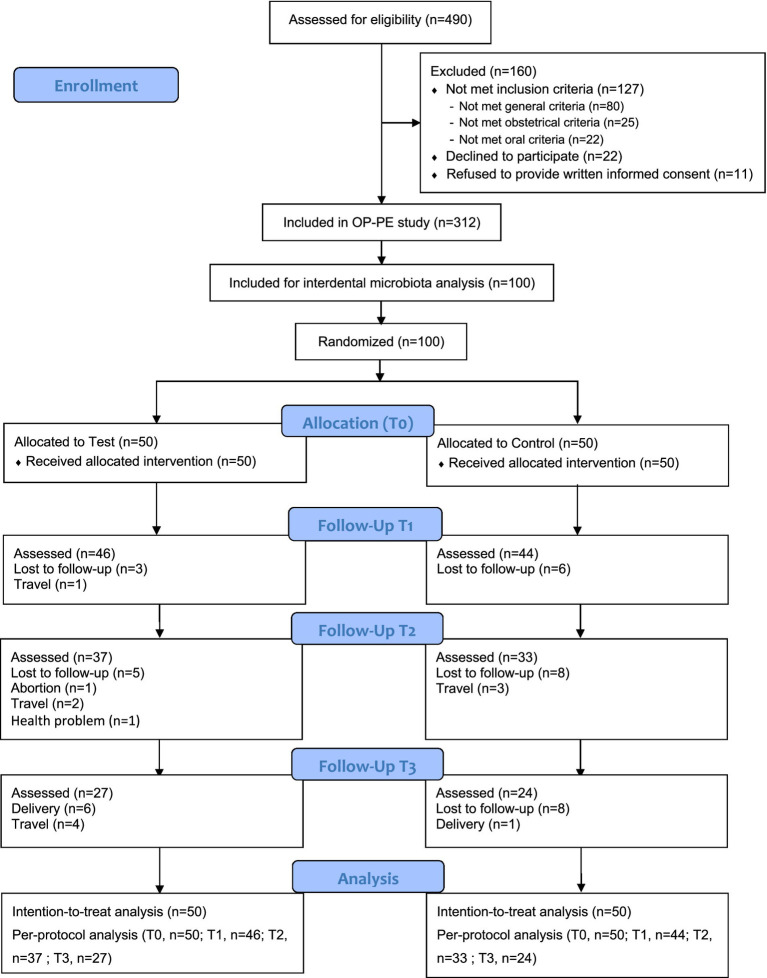
Flowchart of the study. *n*, number of participants.

This trial was conducted between March 2022 and January 2023. Of the 490 eligible pregnant women screened, 330 met the inclusion criteria, and 100 were randomized for interdental microbiota analysis. These participants constituted the intention-to-treat population (Figure 1).

Baseline characteristics between the test and control groups were described in Table 1 and Supplementary Table S7. The mean age of participants was 23.70 ± 4.60 years, ranging from 18 to 37 years. Out of 49 participants lost to follow-up at T3 (23 in test group vs. 26 in control group), 23 were due to documented reasons (e.g., premature delivery, unplanned travel), while 16 (9 in test group vs. 12 in control group) were lost without any provided explanation. The difference between the groups in unexplained losses was not statistically significant (*p* > 0.05). At T1, 90/100 samples (90%) were directly observed and 10/100 (10%) were imputed; at T2, 70/100 (70%) were observed and 30/100 (30%) imputed; and at T3, 51/100 (51%) were observed and 49/100 (49%) imputed (Supplementary Table S9). Imputation proportions were similar between intervention and control groups at each visit. The average compliance rate with interdental brush use in the test group, based on participant logbooks, was 95% over the full study period.

**Table 1:** Baseline characteristics of the study groups (intention-to-treat analysis).

Variable	Test	Control
Age (years)	*N* = 50	*N* = 50
Mean ± SD	24.54 ± 5.08	22.78 ± 3.96
Median (min; max) [IQR]	24 (18; 37) [20–27]	22 (18; 37) [19–27]
Education level, *n*/*N* (%)		
No	8/50 (16%)	12/50 (24%)
1–6 years	8/50 (16%)	13/50 (26%)
7–12 years	16/50 (32%)	8/50 (16%)
≥13 years	18/50 (38%)	17/50 (34%)
Glycemia (mmol/L)	*N* = 50	*N* = 39
Mean ± SD	4.63 ± 0.78	3.36 ± 1.53
Median (min; max) [IQR]	4.6 (0.93; 6.27) [4.25–5.08]	3.7 (0.42; 5.33) [266–4.48]
Arterial blood pressure, *n*/*N* (%)	*N* = 50	*N* = 50
Normal	27/50 (54%)	34/50 (68%)
High	2/50 (4%)	0/50 (0%)
At risk	21/50 (42%)	2/50 (32%)
Platelets (Giga/L)	*N* = 49	*N* = 39
Mean ± SD	295.4 ± 63.94	304.97 ± 70.98
Median (min; max) [IQR]	282 (137; 478) [254–333]	309 (168; 495) [264–345]
Uricemia (mg/L)	*N* = 49	*N* = 35
Mean ± SD	27.3 ± 7.91	19.2 ± 8.16
Median (min; max) [IQR]	27 (10; 45) [23–32]	19 (0; 45) [22–32]
C-reactive protein (mg/dL)	*N* = 48	*N* = 38
Mean ± SD	1.08 ± 1.64	1.20 ± 1.65
Median (min; max) [IQR]	0.1 (0; 10) [0.06–0.94]	0.6 (0; 12) [0–0.71]

Values are reported at baseline prior to intervention.

### 3.2 Change in the interdental total bacteria load during the pregnancy

**Figure 2:**
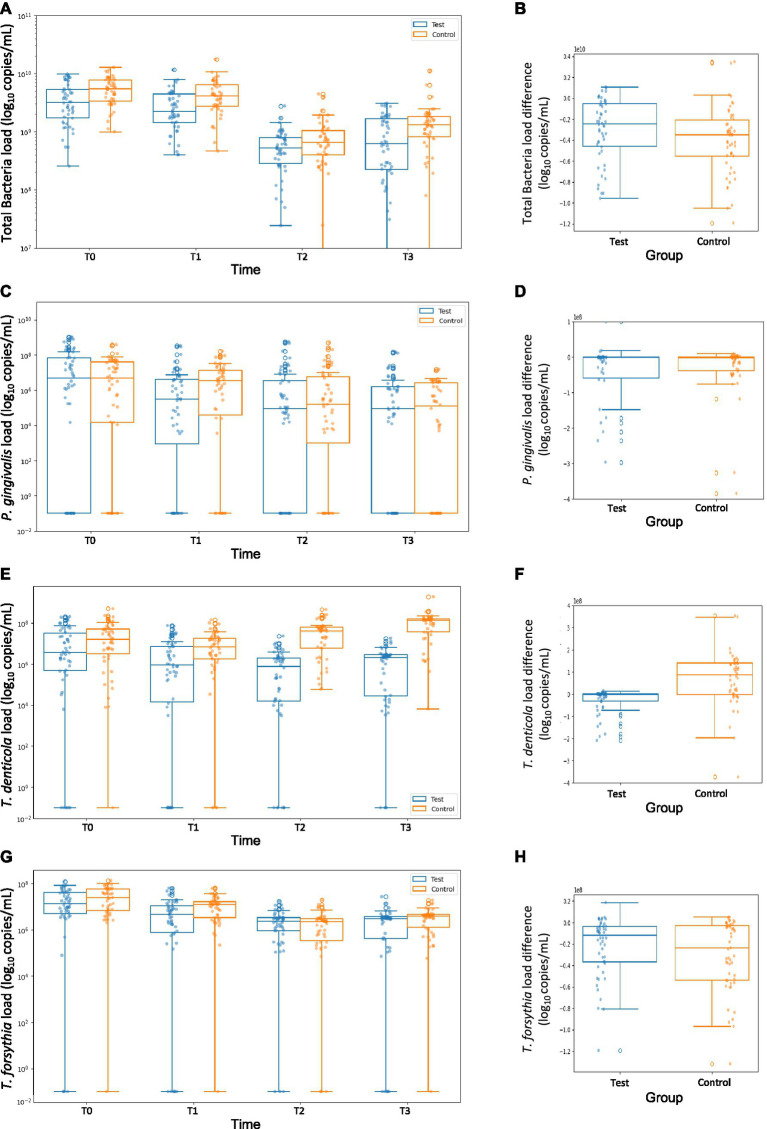
Evolution of total bacteria, *Porphyromonas gingivalis*, *Treponema denticola*, and *Tannerella forsythia* within cohorts (intention-to-treat analysis). **(A)** Evolution of total bacteria. **(B)** Total bacteria load difference between time 0 (baseline, 3 months of pregnancy) and time 3 (8 months of pregnancy). **(C)** Evolution of *Porphyromonas gingivalis*. **(D)** *Porphyromonas gingivalis* load difference between time 0 and time 3. **(E)** Evolution of *Treponema denticola*. **(F)** *Treponema denticola* load difference between time 0 and time 3. **(G)** Evolution of *Tannerella forsythia*. **(H)** *Tannerella forsythia* load difference between time 0 and time 3. Bacterial loads are expressed in log_10_ copies/mL.

The interdental total bacterial load continuously decreased from T0 to T1 and T1 to T2 but increased between T2 and T3 for both the test and control groups (Figure 2). At T3, the median total bacterial load was significantly lower compared to T0 in both groups (*p* < 0.001, Table 2). Absolute log10 changes between T0 and T3 for each bacterial species are reported in Supplementary Table S10. The test group consistently showed lower bacterial loads than the control group at T1, T2, and T3.

**Table 2:** Interdental microbiota load evolution for the test and control groups (intention-to-treat analysis).

Variable	Test	Control	*p*-value^a^
Total bacteria			
T0	*N* = 50	*N* = 50	
Median [IQR]	9.50 [9.24–9.73]	9.74 [9.53–9.89]	0.246
T1	*N* = 50 (ID = 4)	*N* = 50 (ID = 6)	
Median [IQR]	9.35 [9.16–9.64]	9.62 [9.44–9.81]	0.001
% decrease T0-T1 mean [90%CI]	−24.54 [−25.56 to −23.81]	−16.13 [−19.34 to −13.60]	
*p*-value^b^	0.035	0.101	
T2	*N* = 50 (ID = 13)	*N* = 50 (ID = 17)	
Median [IQR]	8.72 [8.45–8.90]	8.82 [8.60–9.02]	0.148
% decrease T1-T2 mean [90%CI]	−79.65 [−79.74 to −79.58]	−81.48% [−83.21% to −80.21%]	
*p*-value^b^	<0.001	<0.001	
T3	*N* = 50 (ID = 23)	*N* = 50 (ID = 26)	
Median [IQR]	8.79 [8.35–9.23]	9.11 [8.91–9.26]	0.010
% decrease T2-T3 mean [90%CI]	56.30 [47.58–62.31]	81.92 [75.31–86.04]	
*p*-value^b^	0.040	<0.001	
% decrease T0-T3 mean [90%CI]	−75.99% [−38.67% to −16.46%]	−71.74 [−76.26% to −68.19%]	
*p*-value^b^	<0.001	<0.001	
% difference Test/Control [90%CI]	−39.63% [−41.07% to −37.21%]		
MLM *p*-value^c^	<0.001		
*Porphyromonas gingivalis*			
T0	*N* = 50	*N* = 50	
Median [IQR]	6.67 [−1.00–7.83]	6.70 [4.17–7.59]	0.689
T1	*N* = 50 (ID = 4)	*N* = 50 (ID = 6)	
Median [IQR]	5.48 [2.94–6.60]	6.52 [4.60–7.1]	0.061
% decrease T0-T1 mean [90%CI]	−79.58 [−89.63 to −76.38]	−55.01 [−56.28 to −50.91]	
*p*-value^b^	0.002	0.009	
T2	*N* = 50 (ID = 13)	*N* = 50 (ID = 17)	
Median [IQR]	4.95 [−1.00 6.53]	5.19 [2.99–6.76]	0.881
% decrease T1-T2 mean [90%CI]	59.05 [47.72–60.64]	67.48 [−40.09–104.82]	
*p*-value^b^	0.387	0.266	
T3	*N* = 50 (ID = 23)	*N* = 50 (ID = 26)	
Median [IQR]	4.94 [−1.00–6.19]	5.08 [−1.00–6.43]	0.582
% decrease T2-T3 mean [90%CI]	−72.20 [−72.67 to −68.51]	−93.74 [−94.98 to −81.60]	
*p*-value^b^	0.071	0.350	
% decrease T0-T3 mean [90%CI]	−90.97 [−95.18 to −89.63]	−95.28 [−95.50 to −94.59]	
*p*-value^b^	<0.001	<0.001	
% difference Test/Control mean [90%CI]	114.25% [98.08–165.12%]		
MLM *p*-value^c^	0.009		
*Treponema denticola*			
T0	*N* = 50	*N* = 50	
Median [IQR]	6.56 [5.67–7.50]	7.21 [6.49–7.71]	0.441
T1	*N* = 50 (ID = 4)	*N* = 50 (ID = 6)	
Median [IQR]	5.94 [4.15–6.86]	6.85 [6.25–7.25]	0.001
% decrease T0-T1 mean [90%CI]	−73.58 [−76.59 to −72.32]	−65.06 [−65.68- −63.57]	
*p*-value^b^	0.005	0.005	
T2	*N* = 50 (ID = 13)	*N* = 50 (ID = 17)	
Median [IQR]	5.88 [4.19–6.30]	7.61 [6.77–7.80]	<0.001
% decrease T1-T2 mean [90%CI]	−75.83 [−76.14 to −74.97]	232.25% [222.25–254.87]	
*p*-value^b^	0.018	<0.001	
T3	*N* = 50 (ID = 23)	*N* = 50 (ID = 26)	
Median [IQR]	6.32 [4.44–6.44]	8.15 [7.56–8.20]	<0.001
% decrease T2-T3 mean [90%CI]	23.27 [12.18–53.20]	166.69 [137.69–180.82]	
*p*-value^b^	0.097	<0.001	
% decrease T0-T3 mean [90%CI]	−92.13 [−92.59 to −91.02]	209.60 [207.32–210.54]	
*p*-value^b^	<0.001	<0.001	
% difference Test/Control mean [90%CI]	−98.38% [−98.48% to −98.14%]		
MLM *p*-value^c^	<0.0001		
*Tanerella forsythia*			
T0	*N* = 50	*N* = 50	
Median [IQR]	7.14 [6.72–7.61]	7.40 [6.83–7.76]	0.492
T1	*N* = 50 (ID = 4)	*N* = 50 (ID = 6)	
Median [IQR]	6.67 [5.89–7.04]	7.09 [6.53–7.23]	0.005
% decrease T0-T1 mean [90%CI]	−61.85 [−67.16 to −58.71]	−59.47 [−60.46 to −56.84]	
*p*-value^b^	<0.001	<0.001	
T2	*N* = 50 (ID = 13)	*N* = 50 (ID = 17)	
Median [IQR]	6.37 [5.96–6.52]	6.36 [5.54–5.49]	0.332
% decrease T1-T2 mean [90%CI]	−69.27 [−71.32 to −64.91]	−80.79% [−82.72 to −79.61]	
*p*-value^b^	<0.001	<0.001	
T3	*N* = 50 (ID = 23)	*N* = 50 (ID = 26)	
Median [IQR]	6.50 [5.62–6.56]	6.58 [6.11–6.66]	0.014
% decrease T2-T3 mean [90%CI]	10.29 [4.88–13.41]	46.72 [35.87–67.60]	
*p*-value^b^	0.509	<0.001	
% decrease T0-T3 mean [IQR]	−87.07 [−87.91 to −86.57]	−88.58 [−88.60 to −88.55]	
*p*-value^b^	<0.001	<0.001	
% difference Test/Control mean [90%CI]	−16.54% [90%CI −25.53 to −10.72]		
MLM *p*-value^c^	<0.0001		

Percentage changes are calculated from log10-transformed values. A reduction of 1 log10 corresponds to a tenfold decrease in bacterial load. Data are expressed in log_10_ copies or in % for the % of variation calculated with values expressed in log_10_ copies/mL. ^a^Mann-Whitney test of the differences with a unilateral alternative hypothesis (H1 test < control). ^b^Wilcoxon rank signed test of the differences. ^c^Mixed linear model of the concentrations over time with a unilateral alternative hypothesis (H1 test < Control). ID, imputation data.

A significant reduction in bacterial load was observed in the test group from T0 to T1 (*p* = 0.035), while no significant change was found in the control group during the same interval. Both groups exhibited a significant decrease between T1 and T2, followed by a significant increase between T2 and T3. At T3, the percentage mean difference in bacterial load reduction (log10 copies/mL) between the test and control groups was −36.63% (95% CI: −41.07% to −37.21%). The per-protocol analysis yielded similar results (Supplementary Table S7; Supplementary Figure S1). Similarly, the two-tailed analysis produced comparable findings (Supplementary Table S8).

### 3.3 Change in the interdental *Porphyromonas gingivalis* load during the pregnancy

The median *P. gingivalis* load decreased steadily from T0 to T3 in both groups (Figure 2). At T3, the median load was significantly lower compared to T0 for both groups (*p* < 0.001, Table 2). While the test group showed lower bacterial loads than the control group at T1 (*p* = 0.061), T2 (*p* = 0.881), and T3 (*p* = 0.582), these differences were not statistically significant.

A significant reduction in *P. gingivalis* load was observed from T0 to T1 in both groups. Decreases were also noted between T1 and T2 and between T2 and T3 in both groups. The percentage mean difference in bacterial load reduction (log10 copies/mL) between the test and control groups at T3 was 114.25% (95% CI: 98.08 to 165.12%). Per-protocol analysis confirmed these findings (Supplementary Table S7; Supplementary Figure S1) and two-tailed analysis led to similar results (Supplementary Table S8).

### 3.4 Change in the interdental *Treponema denticola* load during the pregnancy

Among the targeted pathogens of Socransky’s red complex, *T. denticola* exhibited the most pronounced reduction in the test group. By T3, a significant decrease of −98.38% (95% CI: −98.48% to −98.14%).

The median *T. denticola* load decreased from T0 to T2 in the test group but slightly increased between T2 and T3. In the control group, the load decreased from T0 to T1 but increased thereafter (Figure 2). At T3, the median *T. denticola* load was significantly lower than T0 in the test group (*p* < 0.001) and significantly higher in the control group (*p* < 0.001, Table 2).

The test group showed significantly lower *T. denticola* loads than the control group at T1 (*p* = 0.001), T2 (*p* < 0.001), and T3 (*p* < 0.001). Significant reductions were observed in the test group between T0 and T1 and between T1 and T2, while significant increases were seen in the control group across all intervals. At T3, the percentage mean difference in bacterial load reduction (log10 copies/mL) between the test and control groups was −98.38% (95% CI: −98.48% to −98.14%). Per-protocol analysis confirmed these results (Supplementary Table S2; Supplementary Figure S1) and two-tailed analysis led to similar results (Supplementary Table S8).

### 3.5 Change in the interdental *Tanerella forsythia* load during the pregnancy

At T3, the median load was significantly lower compared to T0 for both groups (*p* < 0.001, Table 2). The median *T. forsythia* load decreased from T0 to T2 and slightly increased between T2 and T3 in both groups (Figure 2).

The test group exhibited significantly lower *T. forsythia* loads than the control group at T1 (*p* = 0.005) and T3 (*p* = 0.014). Significant reductions in *T. forsythia* load were observed from T0 to T1 and T1 to T2 for both groups. At T3, the percentage mean difference in bacterial load reduction (log10 copies/mL) between the test and control groups was −16.54% (95% CI: −25.53% to −10.72%). These findings were consistent with the per-protocol analysis (Supplementary Table S2; Supplementary Figure S1) and two-tailed analysis led to similar results (Supplementary Table S8).

### 3.6 Evolution of oral and obstetrical parameters during the pregnancy

The test group showed significantly better oral health outcomes compared to the control group throughout pregnancy (Table 3). At baseline, oral health parameters, including bleeding on interdental brushing and gingival index, were not significantly different between the two groups, except for the plaque index, which was lower in the test group (*p* = 0.028). Over time, improvements in bleeding on interdental brushing (*p* < 0.001), gingival index (*p* = 0.031 to 0.009), and plaque index (*p* = 0.002 to 0.007) were observed in the test group, with significant differences maintained at T1, T2, and T3. The plaque index increases over time in the control group, whereas it remains stable in the test group. In contrast, no major differences were found between groups for obstetrical parameters such as body mass index, uterine height, or blood pressure.

**Table 3:** Evolution of obstetrical and oral data in test and control groups.

Variable	Test		Control		*p*-value^a^
	*N*	median [IQR]	*N*	median [IQR]	
T0					
Week of pregnancy	50	12 [12–13]	50	12 [12–12]	0.011
Body mass index (kg/m^2^)	50	23.00 [21.24–27.30]	50	22.06 [20.20–25.10]	0.097
Uterine height (cm)	50	11.0 [10.0–12.0]	50	10.0 [9.5–11.0]	0.297
Systolic blood pressure	50	112.0 [104.75–120.25]	50	111.0 [105.0–120.5]	0.655
Diastolic blood pressure	50	75.0 [69.75–80.25]	50	69.0 [65.0–74.0]	0.006
Bleeding on interdental brushing (%)	50	0.53 [0.39–0.8]	50	0.7 [0.56–0.84]	0.059
Gingival index	50	0.23 [0.0–0.64]	50	0.3 [0.0–0.83]	0.713
Plaque index	50	0.33 [0.15–0.68]	50	0.63 [0.14–1.04]	0.028
T1					
Week of pregnancy	46	16 [16–17]	44	16 [15–17]	0.007
Body mass index (kg/m^2^)	46	23.95 [21.11–27.03]	44	22.46 [20.16–25.26]	0.745
Uterine height (cm)	46	14.98 [14.38–15.58]	44	14.00 [12.00–16.04]	<0.001
Systolic blood pressure	46	111.17 [104.33–120.50]	44	111.00 [106.67–118.67]	0.460
Diastolic blood pressure	46	73 [69.25–77.67]	44	70.00 [64.00–74.67]	0.024
Bleeding on interdental brushing (%)	46	0.15 [0.07–0.25]	44	0.74 [0.59–0.89]	<0.001
Gingival index	43	0.00 [0.00–0.50]	42	0.36 [0.00–0.99]	0.031
Plaque index	43	0.25 [0.04–0.60]	42	0.70 [0.33–1.27]	0.002
T2					
Week of pregnancy	37	26.0 [24.0–27.0]	31	24.0 [24.0–25.0]	0.084
Body mass index (kg/m2)	36	24.30 [20.88–28.00]	30	23.00 [20.02–25.58]	0.195
Uterine height (cm)	37	24.0 [20.0–27.0]	25	22.0 [19.0–24.0]	0.103
Systolic blood pressure	37	110.0 [103.0–119.0]	31	108.0 [100.5–114.0]	0.247
Diastolic blood pressure	37	71.0 [65.0–75.0]	31	68.0 [63.5–78.0]	0.698
Bleeding on interdental brushing (%)	37	0.07 [0.0–0.19]	33	0.85 [0.62–0.96]	<0.001
Gingival index	37	0.0 [0.0–0.39]	29	0.64 [0.04–1.0]	0.001
Plaque index	37	0.29 [0.07–0.64]	29	0.68 [0.39–1.04]	0.009
T3					
Week of pregnancy	27	34.0 [33.0–37.0]	20	33.0 [31.75–37.0]	0.180
Body mass index (kg/m^2^)	27	24.20 [22.45–29.50]	20	23.8 [23.08–27.05]	0.830
Uterine height (cm)	27	29.00 [27.50–31.50]	20	28.0 [25.75–30.25]	0.236
Systolic blood pressure	27	115.0 [103.5–120.0]	20	110.5 [106.0–126.5]	0.991
Diastolic blood pressure	27	72.00 [66.50–76.00]	20	67.5 [64.75–74.25]	0.413
Bleeding on interdental brushing (%)	27	0.04 [0.00–0.18]	24	0.74 [0.67–0.89]	<0.001
Gingival index	27	0.00 [0.00–0.39]	20	0.80 [0.00–1.15]	0.009
Plaque index	27	0.39 [0.02–0.68]	20	0.91 [0.50–1.16]	0.007

## 4 Discussion

This RCT assessed the impact of calibrated IDBs on the interdental microbiota of pregnant women, specifically targeting total bacterial load and three key pathogens of Socransky’s red complex: *P. gingivalis, T forsythia,* and *T. denticola*. These pathogens have been reported to be abundant in previous culture-based studies on pregnant women and are strongly associated with pregnancy-related conditions such as pregnancy gingivitis (5) and preeclampsia (23). These pathogens, recognized for their ability to modulate host inflammatory responses, are strongly associated with pregnancy complications such as preeclampsia (7). Recent review has further synthesized the biological and clinical evidence linking periodontitis to adverse pregnancy outcomes, emphasizing mechanisms such as systemic dissemination of periodontal pathogens, endotoxemia, and inflammation-mediated placental dysfunction (24). While causality remains debated, the convergence of epidemiological and mechanistic data strengthens the biological plausibility of an association between periodontal dysbiosis and obstetric risk. Indeed, as key modulators of host inflammatory responses, the reduction of periodontal pathogens may provide mechanistic insight into pathways that have been associated with adverse pregnancy outcomes, including preeclampsia and low birth weight (25); however, the present study was not designed to evaluate clinical obstetrical endpoints.

While other species, such as *Aggregatibacter actinomycetemcomitans* and *Fusobacterium nucleatum*, are also part of the periodontal microbiota, their inflammatory and pathogenic potential in obstetric outcomes is less significant. The decision to focus on the red complex pathogens was driven by their high virulence and critical role in severe periodontal inflammation, which has been shown to impact maternal and fetal health outcomes.

This study utilizes microbiological analysis to explore potential mechanistic pathways related to periodontal modulation during pregnancy. These microbiological outcomes should be interpreted as surrogate biological markers rather than clinical endpoints. By focusing on reductions in major key periodontal pathogens, the study aims to explore how targeted microbiological changes might contribute to improved oral and systemic health during pregnancy. The rationale behind this hypothesis is grounded in the established role of these pathogens in modulating inflammatory responses and their established links to adverse pregnancy outcomes (26).

The observed changes in bacterial load over time reflect the dynamic interplay between physiological changes in pregnancy and the oral microbiome. Our findings suggest that IDBs can modulate the interdental microbiota during pregnancy, with reductions in bacterial load and key pathogens observed in the test group compared to the control group. Among the targeted pathogens of Socransky’s red complex, *T. denticola* exhibited the most pronounced reduction in the test group. By T3, a significant decrease of −98.38% (95% CI: −98.48% to −98.14%) was observed compared to baseline (T0), highlighting the efficacy of IDBs in targeting this highly virulent pathogen. In contrast, the control group demonstrated an increase in *T. denticola* load over the same period, emphasizing the protective role of IDBs.

The results for *T. denticola* were particularly notable, with a substantial reduction of −92.13% observed in the test group by T3, compared to a significant increase of +209.60% in the control group. As a key pathogen strongly associated with periodontal disease, the distinct trajectory of *T. denticola* in the test group highlights the effectiveness of the intervention in controlling its levels. Furthermore, the consistent and significant differences between the test and control groups at all time points underscore the robust impact of interdental cleaning on reducing *T. denticola* load.

Both groups in our trial demonstrated a significant reduction in *P. gingivalis* bacterial load over time, with the test group showing a greater relative reduction. However, the differences between groups at T1, T2, and T3 did not reach statistical significance. This underscores the need for cautious interpretation of the intervention’s effect on *P. gingivalis* and highlights the complexity of managing this pathogen during pregnancy. Although the trend suggests potential benefits from interdental cleaning, the observed effects are significant but modest in magnitude. Additional studies could help confirm these findings in broader populations and refine our understanding of their clinical significance.

In line with the microbiological findings, our clinical data showed that the intervention group experienced consistent reductions in plaque index over the study period, whereas the control group showed a statistically significant increase in plaque index between baseline (T0) and the final follow-up (T3). This divergence suggests a clear protective effect of IDBs on plaque accumulation during pregnancy.

The increase in plaque index in the control group aligns with prior evidence that pregnancy-related hormonal shifts—particularly elevated progesterone and estrogen—can exacerbate gingival inflammation and promote microbial dysbiosis, especially in the absence of effective plaque control (27). The lack of interdental cleaning in the control group likely contributed to biofilm maturation and increased plaque retention in hard-to-reach interdental sites. In contrast, the significant reduction in plaque index in the test group confirms the utility of IDBs as a preventive strategy. This group not only received calibrated IDBs but also benefited from repeated reinforcement of hygiene practices during prenatal visits, which has been shown to improve both knowledge and adherence to oral health behaviors during pregnancy (28).

The three bacteria of the Socransky red complex are known to interact synergistically, which could explain the variability observed in our study. *P. gingivalis* and *T. denticola* mutually enhance their virulence mechanisms to degrade tissue more efficiently. *P. gingivalis* produces growth factors that promote *T. denticola* colonization (29), while *T. denticola* enhances the virulence of *P. gingivalis* through inflammasome signaling. These synergistic interactions, implying gingipains produced by *P. gingivalis,* play a crucial role in the formation of polymicrobial biofilms, contributing to the resistance of these pathogens to mechanical and chemical treatments (30). Similarly, the interaction between *P. gingivalis* and *T. forsythia* modifies the oral environment, although the precise mechanisms behind their interactions have not yet been fully understood. It has been proposed that these mechanisms are mediated through protein–protein interactions (31). Additionally, *T. forsythia* has been demonstrated to inhibit the invasion of *P. gingivalis* into oral epithelial cells (32).

*T. denticola* and *T. forsythia* are frequently found in periodontal biofilms, where they can mutually enhance their resistance to mechanical and chemical treatments (33).

The presence of *P. gingivalis, T. denticola*, and *T. forsythia* in combination can enhance the curriculum and stability of periodontal biofilms (34). This synergy can also intensify the host’s inflammatory response, increasing resistance to therapeutic interventions. *T. denticola* motility may be involved in the synergistic development of biofilms between *P. gingivalis* and *T. denticola* (30).

Importantly, although no major differences were detected in obstetric parameters such as blood pressure, BMI or uterine height between groups, our results are in line with studies suggesting that better periodontal health may have systemic benefits. The lack of observable changes here may be due to sample size limitations (35). However, this sub-study was not powered to detect obstetrical differences. The absence of detectable changes in obstetrical outcomes should not be interpreted as evidence that oral microbiological shifts are purely local; rather, larger trials and/or systemic biomarkers are required to evaluate potential systemic pathways. Further research is essential to determine whether reducing periodontal inflammation contributes to lowering the risk of adverse pregnancy outcomes.

The findings of this study provide a rationale for incorporating interdental hygiene into prenatal care protocols, albeit cautiously. Unlike conventional tooth brushing, which neglects interdental spaces, IDBs specifically target high-risk areas for pathogen accumulation. This tailored approach aligns with the preventive focus of modern maternal healthcare, offering a cost-effective solution for diverse populations, including those in low-resource settings (36). Beyond biological mechanisms, awareness and preventive behaviors play a crucial role in maternal oral health. Recent survey data indicate that many women remain insufficiently informed about the potential relationship between periodontal disease and pregnancy outcomes (37). This gap in awareness highlights the importance of structured educational interventions during prenatal care, reinforcing the relevance of simple and accessible preventive tools such as interdental cleaning. However, additional research is needed to establish the long-term clinical benefits of IDBs in pregnant populations, particularly in relation to systemic health outcomes and the reduction of adverse pregnancy events.

Several limitations warrant consideration. First, microbiological analyses were conducted on a predefined subsample of a larger cohort. Although this approach was prospectively planned, it may have limited statistical power to detect more subtle between-group differences. Second, although some baseline variables differed statistically between groups, these differences were limited in magnitude and remained within clinically comparable ranges. Although baseline diastolic blood pressure differed between groups, oral inflammatory indices were comparable at inclusion; however, residual confounding related to systemic factors cannot be entirely excluded. Moreover, the longitudinal mixed-effects models focused on within-participant changes over time and between-group trajectory differences, thereby minimizing the potential influence of minor baseline imbalances on the primary microbiological outcomes. Third, the study focused on three red complex pathogens. While this targeted approach enhances mechanistic relevance and aligns with the study’s objectives, it does not capture the full complexity of the oral microbiota. Broader microbial analyses may provide a more comprehensive understanding of pregnancy-associated dysbiosis. Fourth, attrition increased over time, particularly by T3, with 28% of participants lost to follow-up. Although intention-to-treat analyses and multiple imputation were applied and sensitivity analyses confirmed the robustness of overall trends, residual bias related to attrition cannot be entirely excluded and may have affected statistical power. Attrition may have reduced statistical power and could affect internal validity, particularly for secondary outcomes. However, consistent findings across intention-to-treat, per-protocol, and sensitivity analyses support the robustness of the primary microbiological outcomes. Fifth, microbiological outcomes represent surrogate markers of periodontal modulation and do not constitute direct evidence of improved obstetrical outcomes. The study was not designed nor powered to detect differences in pregnancy complications, and therefore systemic or obstetric implications remain hypothetical. Sixth, the study was conducted in a specific population, and the results may not be applicable to other demographic groups. Seventh, the observed effects, while statistically significant, are modest in magnitude. This limitation underscores the importance of cautious interpretation of the data and highlights the need for larger, more comprehensive studies to validate these results. Eighth, of the 100 participants initially randomized, 28% were lost with no explanation provided to follow-up by T3. However, to address this attrition, we employed an intention-to-treat analysis complemented by multiple imputation methods for missing data. Sensitivity analyses demonstrated that the overall trends remained consistent regardless of the method used, underscoring the robustness of the findings. However, the impact of attrition on statistical power and potential biases should be acknowledged as a limitation of this study. Ninth, the self-reported nature of compliance data is a limitation, although the very high compliance rate (95%) and regular follow-up at monthly visits suggest that the intervention was followed consistently, reinforcing the validity of the microbiological results. Future studies could incorporate objective adherence measures (e.g., plaque disclosure or digital tracking tools) to improve measurement reliability.

To conclude, this RCT demonstrates the potential of calibrated IDBs to modulate the oral microbiota during pregnancy. By reducing key periodontal pathogens, particularly *T. denticola* and *T. forsythia*, the microbiological findings provide mechanistic evidence of interdental microbiota modulation during pregnancy but do not constitute evidence of systemic or obstetrical benefit. Addressing interdental bacterial load during this critical period of heightened susceptibility to inflammation may help mitigate systemic risks. While the microbiological focus offers mechanistic insights, further research is needed to evaluate how these changes translate into clinical benefits, such as reducing preeclampsia and other adverse outcomes, supporting the integration of interdental hygiene into prenatal care.

## Data Availability

The raw data supporting the conclusions of this article will be made available by the authors, without undue reservation.
