# Supplementary material for: Oral prophylaxis for the reduction of interdental dysbiosis-associated red complex pathogens during pregnancy: a randomized clinical trial
Source: Front Med (Lausanne). 2026 Apr 23;13:1798508. doi: 10.3389/fmed.2026.1798508 (PMC13149147; doi:10.3389/fmed.2026.1798508)
Supplement: Supplementary file 1 [file Data_Sheet_1.docx]

**Oral Prophylaxis for the Reduction of Interdental Dysbiosis-Associated Red Complex Pathogens During Pregnancy: A Randomized Clinical Trial**

Florence Carrouel^1*^, Aida Kanoute^2,3^, Daouda Faye^2^, Maryem Rhanoui^1^, Romain Lan^1,4^, Denis Bourgeois^1^

^1^Laboratory Health Systemic Process (P2S), UR4129, University Claude Bernard Lyon 1, University of Lyon, Lyon, France

^2^Public Health Service, Department of Dentistry, Cheikh Anta Diop University, Dakar 10700, Senegal

^3^Directorate General of Health, Ministry of Health, Dakar, Senegal

^4^Laboratory ADES, Aix Marseille University, CNRS, EFS, Marseille, France,

**Appendix S1**

**Missing data mechanism------------------------------------------------------------------------------------------------** 3

**Sensitivity analysis-------------------------------------------------------------------------------------------------------** 4

**Per-protocol analysis ----------------------------------------------------------------------------------------------------** 6

**CONSORT 2010 checklist --------------------------------------------------------------------------------------------** 11

**List of tables**

**Table S1 – Missing data comparison (MCAR test) ---------------------------------------------------------------** 3

**Table S2 – Complete Case Analysis Results----------------------------------------------------------------------** 4

**Table S3 – Mean Imputation Results-------------------------------------------------------------------------------** 4

**Table S4 – KNN Imputation Results-------------------------------------------------------------------------------** 4

**Table S5 – MICE (Multiple Imputation) Results---------------------------------------------------------------** 4

**Table S6 – Baseline characteristics of the study groups (per-protocol analysis) -----------------------** 6

**Table S7 – Interdental microbiota load evolution for the test and control groups (per-protocol analysis) ------------------------------------------------------------------------------------------------------------------** 7

**Table S8 – Two-tailed sensitivity analyses for between-group comparisons--------------------------------** 8

**Table S9 – Proportion of observed and imputed microbiological data by time point (Intention-to-Treat population) ----------------------------------------------------------------------------------------------------------------** 9

**Table S10 – Absolute log10 median changes between T0 and T3 (intention-to-treat analysis) ---------** 10

**List of figures**

**Figure S1 - Evolution of Total bacteria, *Porphyromonas gingivalis*, *Treponema denticola* and *Tannerella forsythia* for the test and control groups (per-protocol analysis) -----------------** 11

# Missing Data Mechanism

**Table S1 – Missing Data Comparison (MCAR Test)**

|  | Test Variable | T-statistic | P-value |
| --- | --- | --- | --- |
| T1 | T0 | -0.74 | 0.51 |
| T3 | T0 | -0.21 | 0.84 |
| T4 | T0 | -0.14 | 0.89 |

The missing data analysis comparing means between missing and non-missing groups indicated that:

- P-values are all > 0.05, meaning that missingness is not significantly related to the observed variable (T0).
- This indicated that missing data could be considered MCAR.

# Sensitivity Analysis

This Sensitivity Analysis on Missing Data compares different imputation methods:

1. Complete Case Analysis (CCA): Only non-missing data used. This method might introduce bias if missingness is not random.
2. Mean Imputation: Missing values replaced by the column mean. Reduces variability but may distort distributions.
3. KNN Imputation: Predicts missing values using the 5 nearest neighbors. Generally effective but depends on data structure.
4. MICE (Multiple Imputation): Iteratively estimates missing values, producing a more robust imputation.

**Table S2 – Complete Case Analysis Results**

|  | **T0** | **T1** | **T3** |
| --- | --- | --- | --- |
| mean | 4172070000.0 | 2903850000.0 | 503577000.0 |
| std | 2678675908.448053 | 2638670625.8786826 | 299368211.98985034 |
| min | 711750000.0 | 396750000.0 | 49425000.0 |
| max | 9375000000.0 | 11625000000.0 | 1095000000.0 |

**Table S3 – Mean Imputation Results**

|  | **T0** | **T1** | **T3** |
| --- | --- | --- | --- |
| mean | 3997920000.0 | 3022027173.913044 | 560906756.7567568 |
| std | 2848443978.396139 | 2257919360.798346 | 436436784.92530274 |
| min | 253500000.0 | 396750000.0 | 24150000.0 |
| max | 9825000000.0 | 11625000000.0 | 2767500000.0 |

**Table S4 – KNN Imputation Results**

|  | **T0** | **T1** | **T3** |
| --- | --- | --- | --- |
| mean | 3997920000.0 | 3088923000.0 | 588831600.0 |
| std | 2848443978.396139 | 2305286074.155418 | 455277640.75884366 |
| min | 253500000.0 | 396750000.0 | 24150000.0 |
| max | 9825000000.0 | 11625000000.0 | 2767500000.0 |

**Table S5 – MICE (Multiple Imputation) Results**

|  | **T0** | **T1** | **T3** |
| --- | --- | --- | --- |
| mean | 3997920000.0 | 3053425898.9520473 | 564641742.4985979 |
| std | 2848443978.396139 | 2273851792.399911 | 439264931.49856067 |
| min | 253500000.0 | 396750000.0 | 24150000.0 |
| max | 9825000000.0 | 11625000000.0 | 2767500000.0 |

Complete Case Analysis (CCA) removes all observations with missing values and performs the analysis only on the remaining complete cases.

Based on our missing data analysis, the missingness tests (comparison of means between missing and non-missing groups) showed no significant differences (p>0.05; p > 0.05; p>0.05), suggesting that missingness is likely MCAR. Under this assumption, CCA remains a valid and unbiased approach.

# Per-protocol analysis

**Table S6 – Baseline characteristics of the study groups (per-protocol analysis)**

|  | **Actif** | **Control** |
| --- | --- | --- |
| **Age** (years) | N=50 | N=50 |
| mean ± SD | 24.54 ± 5.08 | 22.78 ± 3.96 |
| median (min; max) [IQR] | 24 (18 ; 37) [20 - 27] | 22 (18 ; 37) [19 - 27] |
| **Week of pregnancy** | N=50 | N=50 |
| mean ± SD | 12.4 ± 0.64 | 12.08 ± 0.60 |
| median (min; max) [IQR] | 12 (11 ; 14) [12 - 13] | 12 (11 ; 14) [12 - 12] |
| **Body mass index** (kg/m^2^), | N=50 | N=50 |
| mean ± SD | 24.35 ± 5.25 | 22.90 ± 4.42 |
| median (min; max) [IQR] | 23 (17 ; 40) [21 - 27] | 22 (16 ; 32) [20 - 25] |
| **Education level,** n/N (%) |  |  |
| No | 8/50 (16%) | 12/50 (24%) |
| 1–6 years | 8/50 (16%) | 13/50 (26%) |
| 7–12 years | 16/50 (32%) | 8/50 (16%) |
| ≥13 years | 18/50 (38%) | 17/50 (34%) |
| **Glycemia** (mmol/L) | N=50 | N=39 |
| mean ± SD | 4.63 ± 0.78 | 3.36 ± 1.53 |
| median (min; max) [IQR] | 4.6 (0.93 ; 6.27) [4.25 – 5.08] | 3.7 (0.42 ; 5.33) [266 – 4.48] |
| **Arterial blood pressure,** n/N (%) | N=50 | N=50 |
| Normal | 27/50 (54%) | 34/50 (68%) |
| High | 2/50 (4%) | 0/50 (0%) |
| At risk | 21/50 (42%) | 2/50 (32%) |
| **Platelets** (Giga/L) | N=49 | N=39 |
| mean ± SD | 295.4 ± 63.94 | 304.97 ± 70.98 |
| median (min; max) [IQR] | 282 (137 ; 478) [254 - 333 | 309 (168 ; 495) [264 - 345] |
| **Uricemia** (mg/L) | N=49 | N=35 |
| mean ± SD | 27.3 ± 7.91 | 19.2 ± 8.16 |
| median (min; max) [IQR] | 27 (10 ; 45) [23 - 32] | 19 (0 ; 45) [22 - 32] |
| **C-reactive protein** (mg/dL) | N=48 | N=38 |
| mean ± SD | 1.08 ± 1.64 | 1.20 ± 1.65 |
| median (min; max) [IQR] | 0.1 (0 ; 10) [0.06 – 0.94] | 0.6 (0 ; 12) [0 – 0.71] |
| **Bleeding on interdental brushing** (%) | N=50 | N=50 |
| mean ± SD | 0.36 ± 0.28 | 0.70 ± 0.26 |
| median (min; max) [IQR] | 0.36 (0.00 ; 0.96) [0.12 – 0.52] | 0.80 (0.00 ; 0.96) [0.13 – 0.53] |
| **Plaque index** | N=50 | N=50 |
| mean ± SD | 0.45 ± 0.38 | 0.69 ± 0.54 |
| median (min; max) [IQR] | 0.33 (0.00 ; 1.27) [0.15 – 0.68] | 0.63 (0.00 ; 2.04) [0.14 – 1.04] |
| **Clinical attachment loss** (mm) | N=50 | N=50 |
| mean ± SD | 1.32 ± 1.04 | 2.64 ± 0.62 |
| median (min; max) [IQR] | 1.61 (0.00 ; 3.38) [0.07 – 2.13] | 2.60 (0.00 ; 3.96) [2.32 – 3.06] |

**Table S7 – Interdental microbiota load evolution for the test and control groups (per-protocol analysis)**

|  | **Test** | **Control** | **p-value^a^** |
| --- | --- | --- | --- |
| **Total bacteria** |  |  |  |
| T0 | N=50 | N=50 |  |
| median [IQR] | 9.50 [9.24 - 9.73] | 9.74 [9.53 - 9.89] | 0.246 |
| T1 | N=46 | N=44 |  |
| median [IQR] | 9.35 [9.16 - 9.64] | 9.63 [9.42 - 9.85] | 0.010 |
| % decrease T0-T1 mean [90%CI] | -30.40 [-38.54 - -25.19] | -12.41 [-10.40 - -13.66] |  |
| p-value^b^ | 0.036 | 0.622 |  |
| T2 | N=37 | N=33 |  |
| median [IQR] | 8.68 [8.42 - 8.88] | 8.84 [8.45 - 9.03] | 0.120 |
| % decrease T1-T2 mean [90%CI] | -82.66 [-79.95 - -84.08] | -81.44 [-83.54 - -80.08] |  |
| p-value^b^ | <0.001 | <0.001 |  |
| T3 | N=27 | N=24 |  |
| median [IQR] | 8.47 [8.30 - 9.11] | 9.02 [8.86 - 9.29] | 0.046 |
| % decrease T2-T3 mean [90%CI] | 63.15 [29.32 - 85.55] | 108.56 [23.30 - 154.19] |  |
| p-value^b^ | 0.352 | 0.123 |  |
| % decrease T0-T3 mean [90%CI] | -80.31 [-84.07 - -77.90] | -66.09 [-81.81 - -56.28] |  |
| p-value^b^ | <0.001 | <0.001 |  |
| % difference Test/Control mean [90%CI] | -56.42 [-52.77 - -58.35] | |  |
| MLM p-value^c^ | <0.001 | |  |
| ***Porphyromonas gingivalis*** |  |  |  |
| T0 | N=50 | N=50 |  |
| median [IQR] | 6.67 [-1.00 - 7.83] | 6.70 [4.17 - 7.59] | 0.599 |
| T1 | N=46 | N=44 |  |
| median [IQR] | 5.26 [-1.00 - 6.53] | 6.37 [4.73 - 7.02] | 0.139 |
| % decrease T0-T1 mean [90%CI] | -95.81 [-103.86 - -94.59] | -46.05 [-74.31 - -35.83v |  |
| p-value^b^ | 0.010 | 0.009 |  |
| T2 | N=37 | N=33 |  |
| median [IQR] | 4.99 [4.18 - 5.78] | 5.37 [4.10 - 6.28] | 0.704 |
| % decrease T1-T2 mean [90%CI] | -59.72 [-137.35 - -68.11] | -79.89 [-112.82 - -75.12] |  |
| p-value^b^ | 0.597 | 0.003 |  |
| T3 | N=27 | N=24 |  |
| median [IQR] | 4.96 [4.46 - 6.07] | 4.74 [3.56 - 5.66] | 0.398 |
| % decrease T2-T3 mean [90%CI] | -9.75 [-26.97 - -7.57] | -46.00 [-44.27 - -45.87] |  |
| p-value^b^ | 0.440 | 0.983 |  |
| % decrease T0-T3 mean [90%CI] | -98.48 [-98.95 - -98.41] | -94.14 [-101.84 - -91.36] |  |
| p-value^b^ | 0.024 | 0.002 |  |
| % difference Test/Control mean [90%CI] | 108.67% [86.51 - 266.99] | |  |
| MLM p-value^c^ | 0.104 | |  |
| ***Treponema denticola*** |  |  |  |
| T0 | N=50 | N=50 |  |
| median [IQR] | 6.56 [5.67 - 7.50] | 7.21 [6.49 - 7.71] | 0.441 |
| T1 | N=46 | N=44 |  |
| median [IQR] | 5.87 [3.96 - 6.61] | 6.63 [6.15 - 7.28] | 0.031 |
| % decrease T0-T1 mean [90%CI] | -75.70 [-85.52 - -73.22] | -76.31 [-64.67 - -78.03] |  |
| p-value^b^ | 0.012 | 0.040 |  |
| T2 | N=37 | N=33 |  |
| median [IQR] | 4.81 [3.73 - 6.18] | 7.05 [6.22 - 7.63] | <0.001 |
| % decrease T1-T2 mean [90%CI] | -73.36 [-78.88 - -72.61] | 262.44 [283.81 - 257.36] |  |
| p-value^b^ | 0.138 | 0.113 |  |
| T3 | N=27 | N=24 |  |
| median [IQR] | 4.47 [3.82 - 6.36] | 7.54 [6.44 - 8.13] | <0.001 |
| % decrease T2-T3 mean [90%CI] | -10.89 [-11.82 - -10.40] | 179.76 [-140.29 - 261.45] |  |
| p-value^b^ | 0.761 | 0.011 |  |
| % decrease T0-T3 mean [90%CI] | -91.78 [-92.04 - -91.72] | 140.21 [-154.63 - 183.79] |  |
| p-value^b^ | 0.003 | 0.860 |  |
| % difference Test/Control mean [90%CI] | -98.39 [-97.82 - - 98.54] | |  |
| MLM p-value^c^ | 0.006 | |  |
| ***Tanerella forsythia*** |  |  |  |
| T0 | N=50 | N=50 |  |
| median [IQR] | 7.14 [6.72 - 7.61] | 7.40 [6.83 - 7.76] | 0.492 |
| T1 | N=46 | N=44 |  |
| median [IQR] | 6.54 [5.83 - 7.05] | 7.00 [6.44 - 7.26] | 0.009 |
| % decrease T0-T1 mean [90%CI] | -72.47 [-84.92 - -66.02] | -59.99 [-59.85 - -60.05 |  |
| p-value^b^ | <0.001 | 0.012 |  |
| T2 | N=37 | N=33 |  |
| median [IQR] | 6.14 [5.47 - 6.57] | 5.82 [5.24 - 6.38] | 0.098 |
| % decrease T1-T2 mean [90%CI] | -65.19 [-45.38 - -69.75] | -88.20 [-93.59 - -85.54] |  |
| p-value^b^ | 0.058 | <0.001 |  |
| T3 | N=27 | N=24 |  |
| median [IQR] | 5.65 [4.95 - 6.50] | 6.05 [5.66 - 6.71] | 0.307 |
| % decrease T2-T3 mean [90%CI] | 34.59 [-14.77 - 55.10] | 76.31 [122.04 - 66.28] |  |
| p-value^b^ | 0.957 | 0.016 |  |
| % decrease T0-T3 mean [I90% CI] | -87.10 [-92.98 - -84.06] | -91.68 [-94.28 - -90.40] |  |
| p-value^b^ | <0.001 | <0.001 |  |
| % difference Test/Control [90% CI] | -15.23 [-27.18 - -9.12] | |  |
| MLM p-value^c^ | 0.179 | |  |
| Data are expressed in log_10_ copies or in % for the % of variation calculated with values expressed in log_10_ copies/mL. ^a^Mann-Whitney test of the differences with a unilateral alternative hypothesis (H1 test < control). ^b^Wilcoxon rank signed test of the differences. ^c^Mixed linear model of the concentrations over time with a unilateral alternative hypothesis (H1 test < Control). ID: imputation data. | | | |

**Table S8 – Two-tailed sensitivity analyses for between-group comparisons**

Two-tailed p-values were obtained by doubling the one-tailed p-values used for the directional hypothesis tests (capped at 1.00). These sensitivity analyses did not alter the interpretation of the main findings.

| **Outcome** | **Time point** | **One-tailed p-value** | **Two-tailed p-value** | **Interpretation changed?** | |
| --- | --- | --- | --- | --- | --- |
| Total bacteria | T1 | 0.001 | 0.002 | No |  |
| Total bacteria | T2 | 0.148 | 0.296 | No |  |
| Total bacteria | T3 | 0.010 | 0.020 | No |  |
| *P. gingivalis* | T1 | 0.061 | 0.122 | No |  |
| *P. gingivalis* | T2 | 0.881 | 1.000 | No |  |
| *P. gingivalis* | T3 | 0.582 | 1.000 | No |  |
| *T. denticola* | T1 | 0.001 | 0.002 | No |  |
| *T. denticola* | T2 | <0.001 | <0.002 | No |  |
| *T. denticola* | T3 | <0.001 | <0.002 | No |  |
| *T. forsythia* | T1 | 0.005 | 0.010 | No |  |
| *T. forsythia* | T2 | 0.332 | 0.664 | No |  |
| *T. forsythia* | T3 | 0.014 | 0.028 | No |  |

**Table S9 – Proportion of observed and imputed microbiological data by time point (Intention-to-Treat population)**

Imputed values were estimated using multiple imputation by chained equations (MICE).

| **Time point** | **Total samples (N)** | **Observed n (%)** | **Imputed n (%)** |
| --- | --- | --- | --- |
| T1 | 100 | 90 (90%) | 10 (10%) |
| T2 | 100 | 70 (70%) | 30 (30%) |
| T3 | 100 | 51 (51%) | 49 (49%) |

**Table S10 – Absolute log10 median changes between T0 and T3 (intention-to-treat analysis)**

Absolute changes were computed as (median at T3 − median at T0) using median log10 copies/mL values reported in Table 2 of the main manuscript (ITT population).

| **Outcome** | **Group** | **Median T0 (log10 copies/mL)** | **Median T3 (log10 copies/mL)** | **Absolute change (T3 − T0)** |
| --- | --- | --- | --- | --- |
| Total bacteria | Test | 9.50 | 8.79 | -0.71 |
| Total bacteria | Control | 9.74 | 9.11 | -0.63 |
| *P. gingivalis* | Test | 6.67 | 4.94 | -1.73 |
| *P. gingivalis* | Control | 6.70 | 5.08 | -1.62 |
| *T. denticola* | Test | 6.56 | 6.32 | -0.24 |
| *T. denticola* | Control | 7.21 | 8.15 | +0.94 |
| *T. forsythia* | Test | 7.14 | 6.50 | -0.64 |
| *T. forsythia* | Control | 7.40 | 6.58 | -0.82 |

**Figure S1 – Evolution of Total bacteria, *Porphyromonas gingivalis*, *Treponema denticola* and *Tannerella forsythia* for the test and control groups (per-protocol analysis)**

(A) Evolution of Total bacteria. (B) Total bacteria load difference between time 0 (baseline, 3 months of pregnancy) and time 3 (8 months of pregnancy). (C) Evolution of *Porphyromonas gingivalis*. (D) *Porphyromonas gingivalis* load difference between time 0 and time 3. (E) Evolution of *Treponema denticola*. (F) *Treponema denticola* load difference between time 0 and time 3. (G) Evolution of *Tannerella forsythia*. (H) *Tannerella forsythia* load difference between time 0 and time 3.

| Section/Topic | Item No | Checklist item | Reported on page No |
| --- | --- | --- | --- |
| Title and abstract | | | |
|  | 1a | Identification as a randomised trial in the title | 1 |
|  | 1b | Structured summary of trial design, methods, results, and conclusions (for specific guidance see CONSORT for abstracts) | 2 |
| Introduction | | | |
| Background and objectives | 2a | Scientific background and explanation of rationale | 3 |
|  | 2b | Specific objectives or hypotheses | 3 |
| Methods | | | |
| Trial design | 3a | Description of trial design (such as parallel, factorial) including allocation ratio | 4-5 |
|  | 3b | Important changes to methods after trial commencement (such as eligibility criteria), with reasons | N/A |
| Participants | 4a | Eligibility criteria for participants | 5 |
|  | 4b | Settings and locations where the data were collected | 4 + Appendix p3 |
| Interventions | 5 | The interventions for each group with sufficient details to allow replication, including how and when they were actually administered | 6+ Appendix p3 |
| Outcomes | 6a | Completely defined pre-specified primary and secondary outcome measures, including how and when they were assessed | 6-7 |
|  | 6b | Any changes to trial outcomes after the trial commenced, with reasons | N/A |
| Sample size | 7a | How sample size was determined | 6 |
|  | 7b | When applicable, explanation of any interim analyses and stopping guidelines | N/A |
| Randomisation: |  |  |  |
| Sequence generation | 8a | Method used to generate the random allocation sequence | 4-5 |
|  | 8b | Type of randomisation; details of any restriction (such as blocking and block size) | 4-5 |
| Allocation concealment mechanism | 9 | Mechanism used to implement the random allocation sequence (such as sequentially numbered containers), describing any steps taken to conceal the sequence until interventions were assigned | 5 + Appendix p3 |
| Implementation | 10 | Who generated the random allocation sequence, who enrolled participants, and who assigned participants to interventions | 5 + Appendix p3 |
| Blinding | 11a | If done, who was blinded after assignment to interventions (for example, participants, care providers, those assessing outcomes) and how | 6 |
|  | 11b | If relevant, description of the similarity of interventions | NA |
| Statistical methods | 12a | Statistical methods used to compare groups for primary and secondary outcomes | 6 |
|  | 12b | Methods for additional analyses, such as subgroup analyses and adjusted analyses | 6 |
| Results | | | |
| Participant flow (a diagram is strongly recommended) | 13a | For each group, the numbers of participants who were randomly assigned, received intended treatment, and were analysed for the primary outcome | 17 |
|  | 13b | For each group, losses and exclusions after randomisation, together with reasons | 17 |
| Recruitment | 14a | Dates defining the periods of recruitment and follow-up | 6 |
|  | 14b | Why the trial ended or was stopped | 6 |
| Baseline data | 15 | A table showing baseline demographic and clinical characteristics for each group | 13 |
| Numbers analysed | 16 | For each group, number of participants (denominator) included in each analysis and whether the analysis was by original assigned groups | 6-8 |
| Outcomes and estimation | 17a | For each primary and secondary outcome, results for each group, and the estimated effect size and its precision (such as 95% confidence interval) | 6-8, 14, 15 |
|  | 17b | For binary outcomes, presentation of both absolute and relative effect sizes is recommended | N/A |
| Ancillary analyses | 18 | Results of any other analyses performed, including subgroup analyses and adjusted analyses, distinguishing pre-specified from exploratory | 6-8, Appendix p5-8 |
| Harms | 19 | All important harms or unintended effects in each group (for specific guidance see CONSORT for harms) | N/A |
| Discussion | | | |
| Limitations | 20 | Trial limitations, addressing sources of potential bias, imprecision, and, if relevant, multiplicity of analyses | 10 |
| Generalisability | 21 | Generalisability (external validity, applicability) of the trial findings | 10-11 |
| Interpretation | 22 | Interpretation consistent with results, balancing benefits and harms, and considering other relevant evidence | 9-11 |
| Other information | | |  |
| Registration | 23 | Registration number and name of trial registry | 4 |
| Protocol | 24 | Where the full trial protocol can be accessed, if available | N/A |
| Funding | 25 | Sources of funding and other support (such as supply of drugs), role of funders | 11 |

# CONSORT 2010 checklist
